# Co‐presence of black soldier fly frass, soil‐biodegradable mulch films, and earthworms: effects on film biodegradation, ecotoxicity, and microbial community

**DOI:** 10.1002/jsfa.70064

**Published:** 2025-07-21

**Authors:** Matteo Francioni, Enrica Marini, Arianna De Bernardi, Paride D'Ottavio, Alessio Ilari, Ester Foppa Pedretti, Daniele Duca, Boakye‐Yiadom Kofi Armah, Chiara Rivosecchi, Marco Appicciutoli, Gianluca Brunetti, Marco Bianchini, Luigi Ledda, Maria Teresa Tiloca, Mario Antonello Deroma, Francesca Tagliabue, Cristiano Casucci, Costantino Vischetti, Filippo Vaccari, Francesca Bandini, Edoardo Puglisi, Paola Antonia Deligios

**Affiliations:** ^1^ Università Politecnica delle Marche Dipartimento di Scienze Agrarie, Alimentari ed Ambientali Ancona Italy; ^2^ Department of Civil, Constructional and Environmental Engineering Sapienza University of Rome Rome Italy; ^3^ Department of Agricultural Sciences University of Sassari Sassari Italy; ^4^ Dipartimento di Scienze e Tecnologie Alimentari per la Sostenibilità della Filiera Agro‐Alimentare Facoltà di Scienze Agrarie Alimentari ed Ambientali, Università Cattolica del Sacro Cuore Piacenza Italy

**Keywords:** Biodegradable plastic, microbiome, soil health, *Eisenia fetida*, Comet Assay, NGS, soil fertility

## Abstract

**Background:**

Soil‐biodegradable mulch films (SBF) are used increasingly to enhance crop yields while addressing soil pollution and disposal issues. Another sustainable practice gaining attention is the use of soil amendments derived from waste, such as insect frass from *Hermetia illucens*. However, the combined effects of these elements on soil ecotoxicity and microbiota remain unclear. To investigate their impact, an incubation experiment was conducted, testing the presence/co‐presence of a commercial SBF (10 g kg^−1^), *H. illucens* frass (50 g kg^−1^), and *Eisenia fetida* earthworms in 250 g of dry soil, maintained at 50% water‐holding capacity.

**Results:**

After 22 days, SBF biodegradation reached 9.5% but increased to 62% with *H. illucens* frass and 52% with *E. fetida*, indicating that frass altered microbial communities and that earthworms enhanced soil perturbation, accelerating SBF breakdown. Ecotoxicological analysis confirmed that the tested SBF was not harmful to *E. fetida*, as no mortality or genotoxic damage was observed. *Hermetia illucens* frass also enhanced earthworm biomass, highlighting its potential as a beneficial soil amendment. The addition of soil‐biodegradable mulch film, *H. illucens* frass, and earthworms altered soil microbial composition and functionality, reducing *α* diversity but enriching decomposition‐ and nutrient‐cycling taxa like Bacillaceae and Lichtheimiaceae, enhancing soil fertility.

**Conclusions:**

These findings highlight potential synergy between insect frass and SBF in promoting soil health and accelerating SBF biodegradation in soil. Future research should focus on optimizing application rates and assessing long‐term effects, with particular attention to the potential phytotoxic effects of frass on crops. © 2025 The Author(s). *Journal of the Science of Food and Agriculture* published by John Wiley & Sons Ltd on behalf of Society of Chemical Industry.

## Introduction

The positive effects of mulching in agriculture are well known and include the moderation of soil temperature, reduction of soil water evaporation, and weed control.^1^, ^2^, ^3^ There are various types of mulching, such as the use of living mulches or soil covering with crop residues,^4^, ^5^ but, in recent decades, the use of plastic films has replaced all others.^6^ This has led to serious pollution problems from soils contaminated with plastics, and soil‐biodegradable plastic films have only recently been developed and commercialized.^7^ Soil‐biodegradable mulch films are designed to be buried at the end of crop cycles, addressing the issue of costly and often impractical film removal and disposal.^8^ The biodegradation of the polymers (mostly polyesters) that compose soil‐biodegradable mulch films under aerobic conditions results in the conversion of polymer carbon into CO_2_ and microbial biomass.^8^ Soil‐biodegradable mulch films are mineralized by enzymes and ubiquitous decomposers (microbes and fungi).^9^ However, questions remain about the varying rates at which these films biodegrade, depending on the different agronomic practices farmers may adopt. For instance, it has been reported that different soil‐tillage practices can influence soil water content,^10^ a crucial element for hydrolysis reactions,^11^ while different crop rotations lead to the incorporation of residues with varying C:N ratios,^12^ and the use of various forms of fertilizers and/or amendments may also play a role as they influence the microbial component of the soil.^13^

Insect frass has been widely studied as a fertilizer and soil improver in various crops^14^ and in relation to different insect species. Several mechanisms of positive interaction in subsequent crops have been identified and can be summarized into four categories: (i) growth promotion through nitrogen sources; (ii) growth promotion through balanced NPK macronutrient sources; (iii) growth promotion through other compounds (sugars, phenols, alkaloids, etc.); and (iv) promotion of resistance or reduction of the effects of diseases or abiotic stresses. Regarding nitrogen supply, this effect appears to be more specific to certain orders, such as Coleoptera, Lepidoptera, and Orthoptera.^15^, ^16^, ^17^, ^18^, ^19^ However, many of the studies have focused on forest crops, including pine, eucalyptus, and red oak. In terms of the supply of complete nutrients, this effect seems to be more related to the orders Blattodea and Coleoptera, with numerous studies focusing on the frass of *Tenebrio molitor* applied to agricultural crops.^20^, ^21^, ^22^, ^23^ When considering the effect of supplying other potentially stimulant compounds, this has been found almost exclusively in species of the orders Lepidoptera and Coleoptera, particularly with the supply of sugars, phenols, and flavonoids.^24^ Finally, in terms of the effect on resistance or reduction of biotic and abiotic stresses, the frass that has demonstrated such effects primarily comes from insects of the orders Lepidoptera and Diptera, with the latter specifically involving the black soldier fly (*Hermetia illucens*).^25^, ^26^

The soil biota consists of a large number of macro‐ and micro‐organisms that represent the living part of the terrestrial ecosystem.^27^ Some of these organisms can be bioindicators as they provide physiological responses that reflect environmental changes.^28^, ^29^, ^30^ International guidelines have recognized the earthworm, *Eisenia fetida*, as the standard organism for ecotoxicological research due to its ability to live in close contact with soil. Soil fauna play a pivotal role in maintaining soil health and functionality by enhancing litter decomposition, nutrient cycling, and soil fertility.^31^, ^32^ The gut microbiota of soil macrofauna is of particular interest due to its symbiotic relationship with the host organisms and its effect on biochemical cycling.^32^ A close link exists between gut microbiome and soil microbial composition and efficiency. The process of digestion through an animal intestine acts as a selective mechanism influencing microbial populations within the soil.^32^, ^33^ In annelids, the intestinal tract is enriched with carbon and nitrogen when in comparison with the surrounding soil, which promotes the development of bacterial phyla such as Actinobacteria, Acidobacteria, Firmicutes, and Proteobacteria.^32^, ^33^ Earthworm activity notably increases the total soil microbial biomass, with higher microbial concentrations found in their casts in comparison with bulk soil.^34^

The use of soil‐biodegradable mulch films combined with insect frass could help address both food‐related challenges and circular economy goals, contributing to several Food and Agriculture Organization (FAO) Sustainable Development Goals.^35^ However, the consequences of the coexistence of these elements in agricultural soils, as well as their potential impact on ecotoxicity, are not yet known. Existing studies have highlighted adverse effects on soil fauna physiology and toxicology under laboratory conditions but comprehensive environmental data are still missing. For instance, insect frass can enhance nutrient availability and positively influence the gut microbiota of soil organisms, thereby promoting soil fertility. Conversely, the incorporation of mulch films that degrade into micro‐ and nano‐plastics might disrupt microbial communities within the soil fauna gut, potentially impeding soil processes and functions. Studying the response of earthworms and microorganisms to soil‐biodegradable plastic and frass represents an important measure of evaluating how these materials might affect soil quality.

This study hypothesized that using *H. illucens* frass as an amendment could stimulate soil microbial activity and, consequently, accelerate the mineralization of soil‐biodegradable mulch films. It is also hypothesized that the presence of earthworms could further accelerate the mineralization of soil‐biodegradable plastics due to their soil‐aerating activity, assuming that soil‐biodegradable mulch films are non‐toxic to them. This study had the following objectives:

- To quantify the biodegradation rate of a commercial soil‐biodegradable mulch film.
- To determine whether biodegradation is accelerated in the presence of *H. illucens* frass or in the presence of *E. fetida*.
- To verify that soil‐biodegradable mulch films and *H. illucens* frass do not cause ecotoxicity.
- To examine any changes in the soil microbiome that occur in the presence of soil‐biodegradable mulch films, *H. illucens* frass, and earthworms, either individually or in combination.

## Materials and Methods

### Soil and tested materials

Soil was sampled in October 2022 at a private farm in Morrovalle, Marche region, Italy (43°17′12.55″ N, 13°36′40.03″ E) from a depth of 0–35 cm, which corresponds to the ploughing depth used by the farm. The farm has been using soil‐biodegradable mulch films for about 15 years. Further details on the soil and farm management are provided in Bianchini *et al*.^10^ The soil had a clay loam texture with 43% sand, 23% silt, and 34% clay, and it was characterized by a water‐holding capacity of 420 g kg^−1^, a pH of 7.33, along with an organic carbon content of 1.33 g kg^−1^ and total nitrogen content of 0.75 g kg^−1^.

The commercial soil‐biodegradable mulch film that is normally used on the farm was sampled before being laid in the field. The film was black and made of Mater‐Bi, a biodegradable and compostable bioplastic commercialized by Novamont (Novara, Italy). Using the dry combustion method with an elemental analyzer (Leco CHN628 Series, LECO Corporation, St Joseph, MI, USA), its carbon content was determined to be 67%. After sampling, the film was cut carefully into pieces of about 2 × 2 mm and then stored in a refrigerator at 4 °C for a few days until the start of the incubation experiment.

The frass used in the study was obtained from a dedicated test within a prototype breeding system, where the temperature and humidity were set at 30 °C and 80% relative humidity, respectively. The bioconversion test, which produced the frass, involved approximately 6000 larvae of *H. illucens* aged 4–6 days. These larvae were housed in polypropylene containers measuring 40 × 60 cm with a height of approximately 23 cm, resulting in a larval density of about 2.5 larvae cm^−2^ and approximately 0.11 larvae cm^−3^. Each larva was fed 100 mg of food per day, comprising a mix of two components: fresh green bean waste as the fresh component and wheat storage waste as the dry component (Supporting Information, Figs. S1 and S2). The two waste components were mixed in a ratio of 3.5:1 to achieve a substrate humidity of approximately 70%. The mixture was then ground twice using a Tiger Shark 100 dual‐axis mill (Fulltech Instruments, Formello, Rome). The entire breeding cycle lasted 12 days, with the diet provided in three phases: initially for 7 days, then for 2 days, and finally for 3 days (Supporting Information, Fig. S3). At the end of the bioconversion phase, the frass was separated from the live larvae using a multimedia sieve with 1, 2, 3, and 5 mm sieves. All fractions containing larvae (sizes between 2 and 3 mm) were removed and sent for various chemical characterizations. The remaining material, consisting of exuviae, unprocessed food, and feces, was considered frass and was subsequently stabilized in the air, with the granulometric fractions kept separate. The stabilization process involved keeping the material well‐ventilated for about 5 days to prevent mold or microbial degradation and to minimize as much as possible the release of ammonia, which is naturally produced by *H. illucens*.^36^, ^37^ After stabilization, the frass with granulometry less than 2 mm and between 3 and 5 mm was combined and sent for biodegradation tests, while the frass with granulometry larger than 5 mm was ground and sent for other tests. The analyses conducted at TresSpec CHN indicate that the ash obtained contained 413 g kg^−1^ total carbon and 40 g kg^−1^ total nitrogen (Leco, Saint Joseph, MI, USA).

In this study, the earthworm *E. fetida* was used as a test organism as it had a short life cycle, was easy to breed under laboratory conditions, and was sensitive to contaminants.^38^ *Eisenia fetida* were purchased from Lombricoltura Bella Farnia (Sabaudia, Italy) and maintained under controlled laboratory conditions at 20 ± 1 °C in an organic compost and fed with oats and organic vegetables, as previously described by De Bernardi *et al*.^39^ and Marini *et al* ^40^ Adults with wet body weights between 420 ± 90 mg were selected and acclimatized for a week in the same soil used for the experiment.

### Incubation experiment and measurements

#### Jars preparation and experimental design

About 10 kg of soil was sieved to 2 mm and brought to 50% of its water holding capacity by carefully adding demineralized water to avoid damaging the soil structure. Then, 250 g of equivalent dry soil was placed in aluminum jars (10 cm in height and 10 cm in diameter), equipped with lids that were perforated to allow minimal gas exchange. The jars were kept in an incubator in the dark at a constant temperature of 25 ± 1 °C, and the soil moisture content was maintained throughout the experiment by adding distilled water with a sprayer every 2–4 days.

The following materials were then added to the jars containing 250 g of equivalent dry soil in the selected combinations (treatments): soil (control); soil +50 g kg^−1^ of insect frass (FRS); soil +6 earthworms (EWR); soil +10 g kg^−1^ of soil‐biodegradable mulch film (SBF); soil +10 g kg^−1^ of microcrystalline cellulose (Cell); soil + FRS + SBF; soil + EWR + FRS; soil + EWR + SBF; and soil + EWR + SBF + FRS. The experimental design was completely randomized, with three replicates and nine treatments, resulting in a total of 27 jars. The incubation experiment varied in duration depending on the parameter analyzed. Carbon dioxide evolution was monitored for 22 days across all treatments and continued for an additional 22 days in selected treatments (i.e., soil; soil + Cell; soil + EWR; soil + SBF; soil + EWR + SBF). Treatments involving earthworms (i.e., soil + EWR; soil + EWR + SBF; soil + EWR + FRS; and soil + EWR + SBF + FRS) were maintained for up to 28 days, after which the earthworms were removed for ecotoxicological testing (see below). To continue detecting CO_2_ evolution for the additional 22 days, six new earthworms were reintroduced to the soil + EWR and soil + EWR + SBF treatments. The treatments, duration of the incubation, and analyses conducted are summarized in Table 1.

**Table 1:** Treatments, duration, and analyses conducted in the experiment.

Treatment	Tested material (g)	CO_2_ (days)	Ecotoxicological test (days)	Microbiome analyses (days)
Soil (control)	‐	44	‐	28
Soil + FRS	12.5	22	‐	28
Soil + EWR	2.51^a^	44	28	28
Soil + SBF	2.5	44	‐	28
Soil + Cell	2.5	44	‐	‐
Soil + FRS + SBF	12.5 + 2.5	22	‐	28
Soil + EWR + FRS	2.49 + 12.5^a^	22	28	28
Soil + EWR + SBF	2.53 + 2.5^a^	44	28	28
Soil + EWR+ SBF + FRS	2.61 + 2.5 + 2.5^a^	22	28	28

Abbreviations: Cell, cellulose; EWR, earthworms; FRS, Frass; SBF, soil biodegradable film.

^a^ The grams reported are the approximate mass of six earthworms placed in each jar.

#### Carbon dioxide evolution and film mineralization

Carbon dioxide flux was measured with an EGM‐4 equipped with the supplied soil respiration chamber (PP Systems, Amesbury, MA, USA), which fit perfectly on the jars. The CO_2_ concentration variation inside each jar was recorded every 10 s for about 15 min. The final slope of CO_2_ concentration was calculated from minute 5 to minute 12 of the 15 min sampling duration to avoid reading biases due to possible disturbances from water vapor and/or soil respiration chamber saturation. The CO_2_ flux was calculated using the following equation:

$$CO_{2}\;\text{flux}=\frac{P\;\left(\mathrm{Vc}-\mathrm{Vp}\right)\;}{R\;T}\;a\;\frac{1}{W}$$

where *P* is the atmospheric pressure, *Vc* is the volume of the camber, *Vp* is the volume of the plastic case, *R* is the ideal gas constant, *T* is the absolute temperature, *a* is the CO_2_ slope, and *W* is the soil weight.

The percentage of the SBF mineralization was calculated according to the standard ISO 23517, 2021.^41^ The mineralization curve was constructed by calculating the area under the cumulative treatment curves using the trapezoidal rule across all treatments. Then, by subtracting the contributions of each material, the CO_2_ evolution from the mineralization of the film was isolated. For example, the percentage of mineralization achieved by the SBF in the Soil + FRS + SBF treatment was calculated by subtracting the area under the curve for the soil (control) and FRS from that of the Soil + FRS + SBF treatment.

#### Ecotoxicological tests with *Eisenia fetida*

After 28 days of exposure, earthworms were taken from each jar to evaluate the ecotoxicological effects of soil‐biodegradable mulch films and frass. All earthworms were weighed to assess the changes in biomass through the growth rate, which is calculated as the percentage of weight change between the experiment's start and end.

One earthworm per replicate (three for each treatment) was employed for the genotoxicity assessment with the Comet Assay. The study followed a validated protocol previously described in the literature.^39^ Briefly, coelomocytes were collected after earthworm extrusion in an extrusion buffer. Then, the immune cells were washed, counted, and stratified with low‐melting agarose on the slides. After solidification, lysis, and DNA unwinding, the electrophoresis phase started. Finally, the slide was stained, and the comet image was acquired with a Lionheart FX Automated Microscope (Biotek, Winooski, VT, USA). The DNA damage index used was tail length.^39^

#### Soil microbiome analysis

Biodiversity analysis of soil microbial communities was based on high‐throughput sequencing of 16S rDNA amplicons for bacteria and internal transcribed spacer (ITS) regions for fungi. Soil samples from all the treatments were collected at time 0 (T0) and after 28 days (T28). Total DNA was extracted from soil samples using Soil Fast DNA Spin Kit for Soil (MP Biomedicals, Irvine, CA, USA) following the manufacturer's protocol. The V3‐V4 region of 16S ribosomal (rRNA) gene was amplified using the universal primers 343F (5′‐TACGGRAGGCAGCAG‐3′) and 802R (5′‐TACNVGGGTWTCTAATCC‐3′), as previously described in detail.^42^, ^43^ Fungal communities were analyzed using the universal primers ITS‐1 (5′‐TCCGTAGGTGAACCTGCGG‐3′) and ITS‐2 (5′‐GCTGCGTTCTTCATCGATGC‐3′) as described by White *et al*.^44^ The sequencing process was performed by Novogene UK (Cambridge, UK), using the TruSeq DNA sample preparation kit for amplicon preparation (REF 15026486, Illumina Inc., San Diego, CA, USA). The Novaseq 6000 Illumina instrument (Illumina Inc.) was used to obtain 250 bp paired‐end reads. Illumina barcode demultiplexing and base calling were performed with MiSeq Control software version 2.3.0.3, RTA v1.18.42.0, and CASAVA v1.8.2.^45^ Raw sequences were aligned with the ‘pandaseq’ script^46^ with a minimum overlap of 30 bp between read pairs and a maximum of two mismatches allowed. After the filtration, trim, denoising of the demultiplexed sequences, the chimeric sequences were recognized and removed using the QIIME 2 vsearch plugin to acquire the feature table of amplicon sequence variants (ASV). The QIIME 2 feature‐classifier plugin was then used to align ASV sequences with pre‐trained Silva (https://www.arb-silva.de/; trimmed to the V3–V4 region using the 338F/806R primers) and UNITE (https://unite.ut.ee/; trimmed to the ITS1–ITS2 region) databases to generate taxonomy tables. Thermal cycling conditions, primer concentrations, and volumes are provided in Supporting Information, Table S1.

### Statistical analysis

The parameters that were analyzed were subjected to various statistical tests based on their specific characteristics. Data were tested for parametric assumptions, and the corresponding non‐parametric test was applied when these assumptions were violated.

A two‐way repeated measures analysis of variance (ANOVA) was conducted to analyze the effects of time (incubation days, within‐subject factor) and treatments (tested materials and their combinations, between‐subject factor) on soil CO_2_ emissions. A one‐way ANOVA followed by a Tukey post‐hoc test was performed on the percentage of mulch film mineralization at days 22 and 44. The CO_2_ statistical analyses were performed using SPSS, version 29 (SPSS Inc., IBM, Chicago, IL, USA) and the complete ANOVA table is available in the Supporting Information (Table S2).

A one‐way ANOVA followed by a Tukey post‐hoc test was conducted to examine the effect of frass and/or mulch films on earthworm growth rate and tail length. For mean earthworm weight and tail length, a Kruskal–Wallis non‐parametric test followed by a Dunn post‐hoc test was used.

Statistical analysis was performed on sequencing data with R software version 4.3 supplemented by Vegan^47^ and MicrobiomeAnalyst software.^48^ Significance was set at 0.05 for all analyses. Both packages were used to produce principal component analysis and taxonomical bar plots, *α* diversity analysis was based on Shannon's index, *β* diversity analysis using principal coordinates analysis (PCoA) of Bray–Curtis distances at the feature level, grouped by treatment. Statistical significance was tested by permutational multivariate analysis of variance (PERMANOVA) with pairwise comparisons while differential abundance analysis was produced using the linear discriminant analysis effect size (LEfSe) method^49^ on bacterial and fungal genera (linear discriminant analysis > 2; *P*‐value <0.05 false discovery rate adjusted).

## Results

### Carbon dioxide evolution and film mineralization

**Figure 1:**
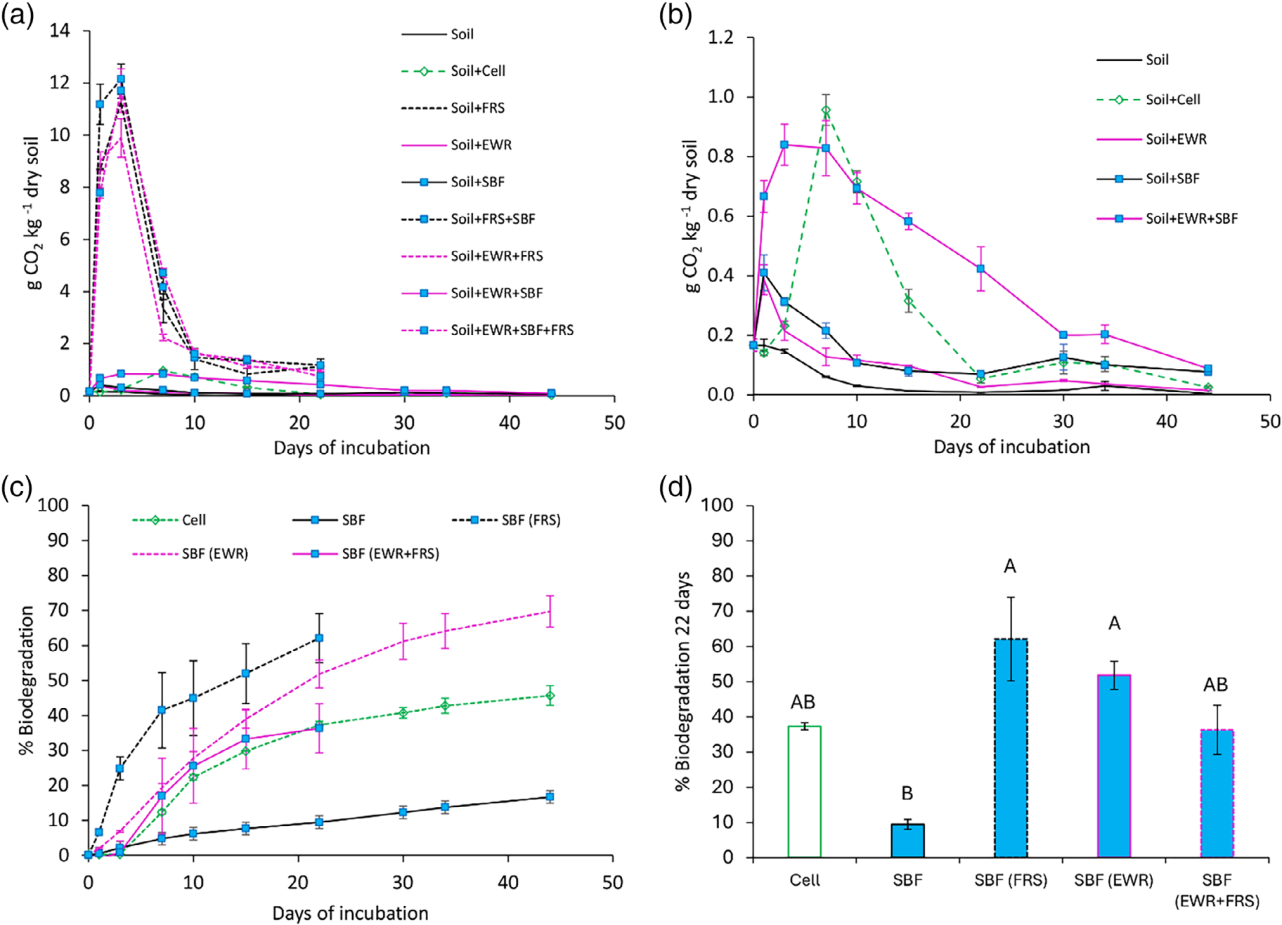
Temporal variations in CO_2_ emissions from combinations of soil (control), cellulose (Cell), frass (FRS), earthworms (EWR), and soil‐biodegradable films (SBF). (a) All treatments. (b) Treatments excluding FRS, to highlight differences among the remaining treatments. (c) Mineralization curves for Cell and all treatments including SBF. (d) Percentage of SBF biodegradation after 22 days in the presence of FRS and EWR, alone or in combination. Parentheses in (c) and (d) indicate co‐presence with SBF. Values represent the means of three replicates; error bars indicate standard errors of the mean. Different lower case letters in panel (d) indicate significant differences (Tukey's HSD test, *P* < 0.05).

Carbon dioxide emissions rapidly increased within 24 h following the addition of all tested materials, with FRS treatments significantly outperforming the others, showing values up to ten times higher (Fig. 1(a),(b)). The peak emission time varied depending on the added material. Both Soil + EWR and Soil + SBF treatments reached their emission peak on the first day of incubation, whereas Soil + EWR + SBF peaked on the third day (Fig. 1(b)). All treatments involving FRS reached their emission peak on the third day, whereas Cell peaked after 7 days (Fig. 1(b)). Following the peak, all treatments exhibited a general decline in CO_2_ emissions, although emissions never completely ceased, even in the control (Fig. 1(b)).

The mineralization curve for SBF appeared stable, and after 22 days of incubation, it did not show a declining phase, unlike the other treatments, including Cell (Fig. 1(c)). At the end of the 22‐day period, approximately 9.5% of the carbon in SBF had been mineralized, whereas the carbon mineralization in Cell reached about 37% (Fig. 1(d)). The presence of FRS enhanced the biodegradation of SBF, resulting in 62% carbon mineralization within 22 days, whereas the presence of EWR led to approximately 52% (Fig. 1(d)). The combined presence of FRS and EWR resulted in lower carbon mineralization compared to the presence of each alone, stabilizing at around 36% after 22 days (Fig. 1(d)). After 44 days of incubation, SBF reached about 17% carbon mineralization, while SBF in the presence of EWR achieved 70% carbon mineralization (Fig. 1(c)).

### Ecotoxicological effects on earthworms

No earthworms escaped or died during the 28‐day experiment. The mean weights of earthworms in each treatment were not statistically different at the start. After 28 days, earthworms exposed to FRS alone or to FRS combined with SBF had a significantly higher mean weight compared to those exposed to SBF alone or to soil alone (Table 2).

**Table 2:** Earthworms' mean weight (g) ± standard error per treatment at the start and end of the experiment. Different lowercase letters represent significant differences between treatments according to the Kruskal–Wallis–Dunn non‐parametric test (*n* = 3, *P* = 0.05).

Treatment	0 Days	28 Days
EWR	0.42 ± 0.11 a	0.26 ± 0.07 b
EWR + FRS	0.42 ± 0.10 a	0.35 ± 0.06 ab
EWR + SBF	0.42 ± 0.07 a	0.27 ± 0.04 b
EWR + FRS + SBF	0.43 ± 0.08 a	0.38 ± 0.06 a

Abbreviations: EWR, earthworms; FRS, frass; SBF, soil biodegradable film.

**Figure 2:**
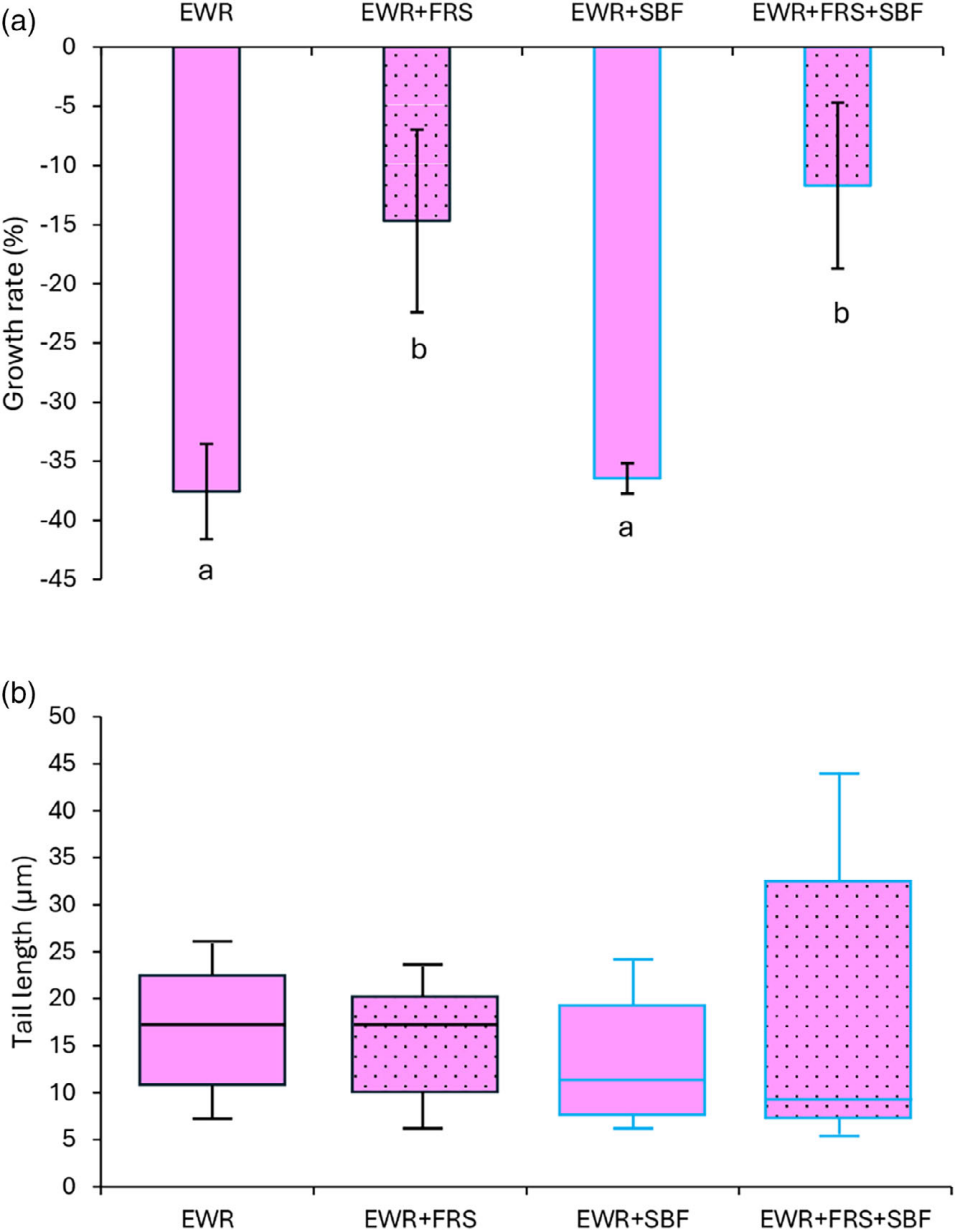
(a) Growth rate of earthworms, calculated as the change in biomass between the start and end of the experiment. Different lower case letters indicate significant differences between treatments according to the ANOVA–Tukey test (*n* = 3, *P* = 0.05). (b) Tail length (μm), an index of DNA damage in earthworm coelomocytes after 28 days of exposure. No significant differences between treatments were detected according to the Kruskal–Wallis–Dunn test (*P* = 0.05). In both panels, values represent the means of three replicates, and error bars indicate the standard error of the mean. Treatment codes: EWR = earthworms, FRS = frass, SBF = soil‐biodegradable film.

To provide a more comprehensive overview, the average weight values were recalculated to determine the growth rate, as shown in Fig. 2(a). A generalized reduction in growth rate was observed across all treatments. Significant weight losses of 37.53% and 36.47% were recorded for the EWR and EWR + SBF treatments, respectively. Earthworms exposed to frass alone or in combination with mulch film showed the smallest decreases in biomass (−14.59% and −11.67%, respectively). The lowest and highest DNA damage values (tail lengths of 5 and 44 μm) were recorded in the treatments with all combinations; despite this, no significant differences were recorded between treatments on day 28 (Fig. 2(b)).

### Effects of frass and mulch films on soil microbiome

**Figure 3:**
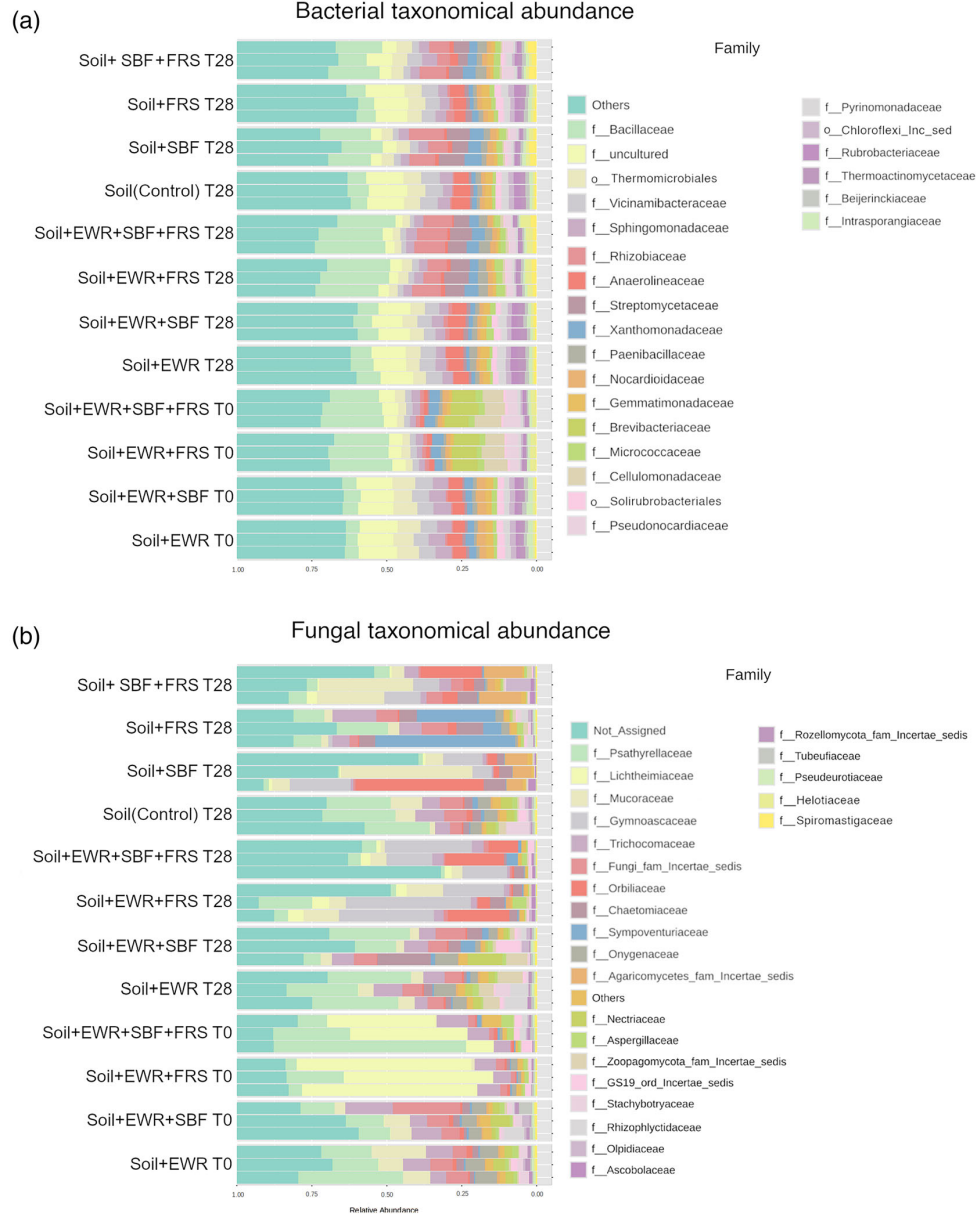
Results of amplicon sequencing analysis. Taxa bar plot displaying the relative abundance of microbial families for (a) bacteria and (b) fungi across all treatments at two time points: T0 and T28. Treatment groups are labeled alongside the bar plots, with specific microbial families indicated for panels (a) and (b). Each family is represented using a distinct color.

The treatments led to significant shifts in both bacterial and fungal communities, especially in treatments involving the addition of *H. illucens* frass and the presence of *E. fetida* (Table 3, Fig. 3).

**Table 3:** Summary of microbial taxa dynamics in response to experimental treatments. Taxa are organized by kingdom, and ordered hierarchically by taxonomic rank (phylum, family, genus). Arrows indicate observed increases (↑) or decreases (↓) in relative abundance under specific treatment conditions and time points.

Kingdom	Taxon	Observed dynamics	Taxonomic rank
Bacteria	Acidobacteria	↓in Soil + EWR + SBF + FRS T0	Phylum
	Actinobacteria	↑ in Soil + EWR + SBF + FRS T0	Phylum
	Chloroflexi	Dominant; ↓ in frass treatments T0	Phylum
	Firmicutes	↓in Soil + EWR + SBF + FRS T0	Phylum
	Bacillaceae	↑ in all frass treatments (T0 and T28)	Family
	Micrococcaceae	↑ at T0 in frass treatments	Family
	Streptomycetaceae	↑ at T28 in all SBF‐treated samples	Family
	Vicinamibacteraceae	↑ in control treatments	Family
	*Bacillus*	↑ in frass + SBF at T28	Genus
	*Brevibacterium*	↑ in frass treatments at T0	Genus
	*Pseudactinotalea*	↑ in frass treatments at T0	Genus
	*Saccharomonospora*	↑ in frass treatments at T0	Genus
	*Sphingomonas*	↑ in control treatments	Genus
	*Streptomyces*	↑ in frass + SBF at T28	Genus
Fungi	Ascomycota	Dominant across treatments	Phylum
	Basidiomycota	Dominant across treatments	Phylum
	Mucoromycota	↑ in frass‐amended treatments T0	Phylum
	Gymnoascaceae	↑ at T28 in frass treatments	Family
	Lichtheimiaceae	↑ in frass‐amended treatments T0	Family
	Sympoventuriaceae	↑ uniquely in Soil + EWR + FRS T28	Family
	*Arachniotus*	↑ at T28 in Soil + EWR + FRS and Soil + EWR + SBF + FRS	Genus
	*Chrysosporium*	↑ at T28 in Soil + EWR + FRS and Soil + EWR + SBF + FRS	Genus

At the bacterial phylum level, all treatments were dominated by Actinobacteria, Proteobacteria, Acidobacteria, Chloroflexi, and Firmicutes (Supporting Information, Fig. S5). Notably, the treatments Soil + EWR + FRS and Soil + EWR + SBF + FRS at T0 showed higher relative abundances of Actinobacteria and lower levels of Acidobacteria and Firmicutes, indicating that the combined application of SBF and frass altered the microbial community structure rapidly (Table 3; Supporting Information, Fig. S5). At the family level, Bacillaceae increased significantly in all frass‐treated samples at both T0 and T28, while Micrococcaceae was notably enriched at T0 in treatments with frass, especially in Soil + EWR + FRS and Soil + EWR + SBF + FRS (Fig. 3). At T28, Streptomycetaceae became more abundant in all SBF‐treated samples, suggesting a shift toward bacterial taxa associated with organic matter degradation and compost‐like environments (Table 3).

For fungi, the dominant phyla across treatments were Ascomycota, Basidiomycota, and Mucoromycota (Table 3; Supporting Information, Fig. S5). Lichtheimiaceae were enriched in frass‐amended treatments at T0 (e.g., Soil + EWR + FRS and Soil + EWR + SBF + FRS), whereas Gymnoascaceae showed higher abundance at T28 in the same treatment groups. Interestingly, the Soil + EWR + FRS T28 treatment displayed a unique increase in Sympoventuriaceae, which was relatively less abundant in other conditions (Table 3). Overall, the fungal community was more heterogeneous than the bacterial community.

**Figure 4:**
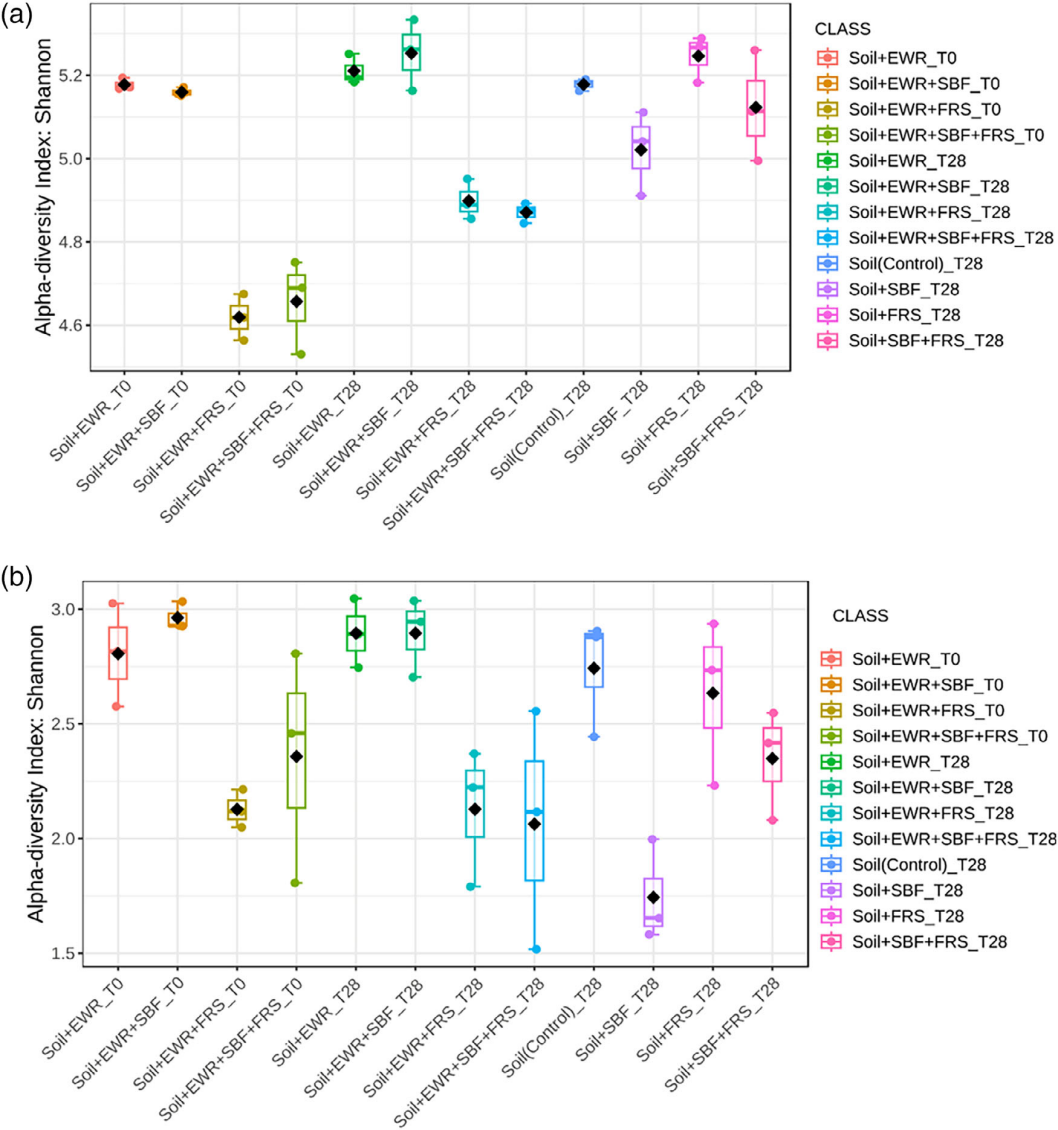
Results from amplicon sequencing analysis. Alpha diversity graph depicting Shannon's diversity index across the treatments for bacteria (a) and fungi (b). Treatment names (classes) are listed to the right of the graph.

Shannon's *α*‐diversity analysis revealed that frass treatments, particularly Soil + EWR + FRS T0 and Soil + EWR + SBF + FRS T0, had the lowest bacterial and fungal diversity. The presence of frass without *E. fetida* (Soil + EWR + SBF T28) also showed a non‐significant downward trend in both bacterial and fungal *α*‐diversity (Fig. 4). Beta‐diversity analyses (Supporting Information, Fig. S4) further highlighted that Soil + EWR + FRS T0 and Soil + EWR + SBF + FRS T0 formed distinct clusters for both bacteria and fungi, indicating early and strong divergence from the other treatments.

**Figure 5:**
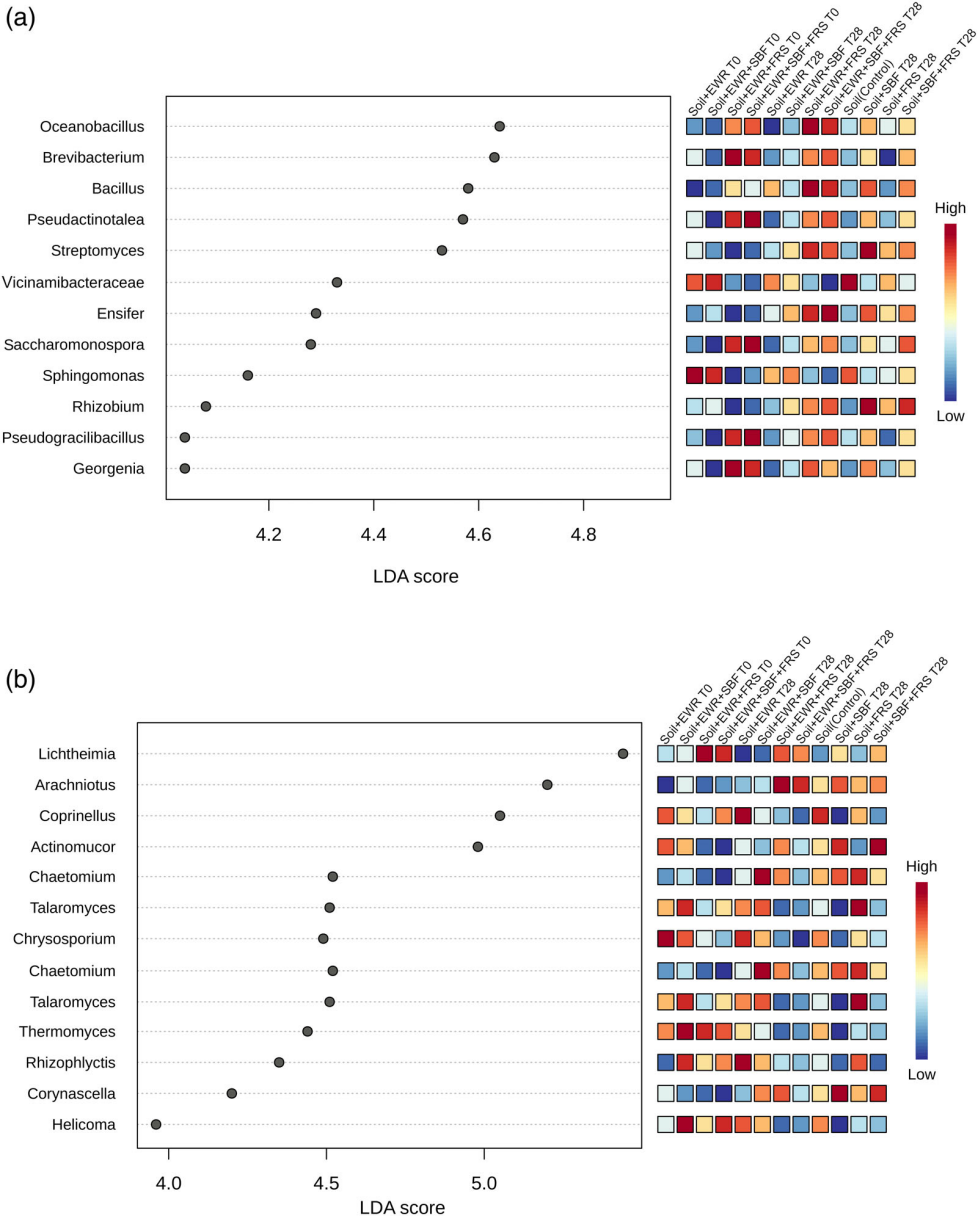
Results from amplicon sequencing analysis: LEfSe analysis highlighting differential abundances of bacterial (a) and fungal (b) genera. The plot displays all genera significantly modulated by the treatments, with a linear discriminant analysis score > 3 and *P* < 0.05 after false discovery rate adjustment.

Linear discriminant analysis effect size analysis identified treatment‐enriched microbial taxa (Fig. 5). For bacteria, frass presence at T0 favored genera such as *Brevibacterium, Saccharomonospora*, and *Pseudactinotalea*, while the addition of both frass and SBF at T28 promoted *Bacillus* and *Streptomyces*, well known decomposer genera. Conversely, *Vicinamibacteraceae* and *Sphingomonas* were enriched in control treatments. For fungi, *Lichtheimia* was enriched significantly in frass treatments at T0, while *Arachniotus* and *Chrysosporium* increased in abundance in Soil + EWR + FRS and Soil + EWR + SBF + FRS at T28. These genera include fast‐growing, thermotolerant saprotrophs commonly found in compost or manure environments. Across all treatments, the bacterial communities were dominated by Actinobacteria, Proteobacteria, Acidobacteria, and Chloroflexi.

## Discussion

### Effects of frass and earthworms on mulch film mineralization

The results support the hypothesis that the addition of *H. illucens* frass and the presence of earthworms both accelerated the biodegradation rate of the mulch film. However, it is likely that the underlying mechanisms driving this acceleration are distinct. It is well established that the biodegradation process of mulch films is complex but can be divided into three fundamental and interconnected steps, which often co‐occur.^8^ Initially, the film is colonized by soil microorganisms naturally present in agricultural soils.^9^ This is followed by enzymatic depolymerization of polymers, carried out by both microorganisms and abiotic hydrolysis. This phase, typically regarded as the rate‐limiting step in polymer biodegradation in soils,^8^, ^50^ can explain the slower biodegradation of mulch films observed in the absence of frass and/or earthworms. The third step involves the assimilation and utilization of the mulch film carbon for microbial energy production and biomolecule synthesis. The addition of insect frass, as observed in other studies,^19^ led to a substantial increase in the mineralization of the labile components of frass carbon, which enhanced microbial biomass but simultaneously reduced microbial biodiversity. This specialization in microbial communities (mostly Actinobacteria and Mucoromycota) enabled the exploitation of the mulch film‐carbon for metabolic processes, thereby increasing its biodegradation. It is well known that *H. illucens* possesses a diverse microbiome that depends significantly on its diet and is only partially retained in the frass.^51^ However, recent studies suggest that the gut microbiota of *H. illucens* can degrade (by surface alteration) even non‐biodegradable polymers such as polystyrene, highlighting its vast potential.^52^

The bioturbation and burrowing activities of earthworms are closely linked to soil health, not only by improving soil structure but also by redistributing organic matter and enhancing soil aeration through burrowing.^53^ The greater degradation of mulch films in the presence of earthworms was largely attributed to increased aeration of the incubated soil. Mineralization (i.e., the conversion of polymer carbon into new microbial biomass, CO_2_, and H_2_O) is an O_2_‐dependent process.^7^, ^8^ An additional potential explanation of higher biodegradation carried out by earthworms is that the physical mixing of soil and mulch films during burrowing probably increased the contact surface area thus enhancing the adherence of mulch film fragments.^10^

### Effects of mulch films and frass on earthworms' ecotoxicology

The hypothesis that soil‐biodegradable mulch films and *H. illucens* frass are non‐toxic to earthworms was confirmed. The fact that no earthworms exposed to both treatments died or escaped during the 28‐day experiment supports this hypothesis, as do similar results reported in other studies. Experiments with *Eisenia andrei* in standard OECD soil using bio‐based plastics composed mainly of Polylactic Acid, Poly(3‐hydroxybutyrate‐co‐3‐hydroxyvalerate), and Poly(butylene adipate‐co‐terephthalate) at concentrations of 1, 5, 25, and 125 g kg^−1^ showed no mortality at any of the concentrations tested.^54^ A study investigated the effects of biodegradable polymers at concentrations of 10 and 25 g kg^−1^ on *E. fetida* and reported a very high survival rate.^55^ Similar findings were observed in another study, with earthworm survival rates exceeding 90% in all tests.^56^

This study also examined the growth rate of *E. fetida* after 28 days of exposure. The results showed a generalized reduction in growth rate, but no significant differences were observed between the control (earthworms in the presence of only soil) and the treatment with soil‐biodegradable mulch film. In line with these results, a study found a reduction in earthworms' body mass in treatments with PLA‐based plastics compared to the control, although these differences were not statistically significant.^54^ Similar results were observed in a study reporting a decrease in growth rate for PLA‐based plastics applied at concentrations of 5, 25, and 50 g kg^−1^; however, these values were not significantly different from the control. The same study also reported a significant weight loss in earthworms exposed to synthetic polymers such as low‐density polyethylene (LDPE) and polyvinyl chloride (PVC). A recent mesocosm study using *E. fetida* in artificial soil exposed to aliphatic‐aromatic copolyester residues from the composting process (at 2 and 7 g kg^−1^) showed a general reduction in growth rate across all samples, although this reduction was not significant. In the present study, the decline in growth rate was more pronounced in the treatments without *H. illucens* frass, suggesting that earthworms use it as a food source. Several recent studies confirm that insect frass is rich in both macro‐ and micronutrients.^14^, ^57^, ^58^, ^59^, ^60^ Indeed, insect frass has been increasingly considered a sustainable resource in cropping systems and a promising alternative to conventional fertilizer. Despite this, information on frass's impact on soil fauna, such as earthworms or microbial communities, has not been investigated extensively.^22^

There is limited literature on the effect of soil‐biodegradable mulch films on ecotoxicology, and only a few studies have investigated the genotoxic damage induced by biodegradable polymers on the non‐target organism *E. fetida*. To investigate these aspects more thoroughly, the Comet assay was performed on the earthworm coelomocytes after 28 days of exposure, with a particular focus on the tail length parameter. The Comet assay is a widely used and effective technique for assessing genotoxicity in environmental biomonitoring, valued for its directness and ease of use.^29^ In the present study, no significant DNA damage was recorded for any of the tested materials at the end of the ecotoxicological trial. Similar results were found in a recent study by Shang *et al*.,^61^ which investigated the ecotoxicological effects of biodegradable microplastics PLA (70 g kg^−1^) and conventional polystyrene applied alone or combined with cadmium. Tail DNA percentages and Olive tail moment values suggested that PLA alone did not induce significant damage to coelomocytes. In contrast, in another experiment a certain DNA damage response was observable in earthworms subjected to bio‐packaging residues added to 7 g kg^−1^ in comparison with controls and treatment with 2 g kg^−1^ residues.^39^ A further study found a significant increase in 8‐hydroxydeoxyguanosine parameter of earthworms exposed to 10 g kg^−1^ PLA; these results reflect the level of DNA oxidative damage.^62^

In contrast with previous studies, no DNA damage was recorded in earthworms in the present study. This can be explained by two factors. First, the ecotoxicity test was conducted in agricultural soil, which, compared to other experiments using artificial soil, may have contributed to buffering the toxicity of the soil‐biodegradable mulch film. In fact, several studies have shown that the toxicity of some contaminants in natural and artificial soils can change due to the different physicochemical properties of the soils.^63^ Second, this response is likely due to the type of material used in the soil‐biodegradable mulch film, which was primarily starch,^7^ even though the concentrations to which the earthworms were exposed might seem relatively low for an ecotoxicological study. These concentrations would be extremely high under open field conditions, as the application of a typical mulch film in soil with a bulk density of 1.5 g cm^−3^ reaches a concentration of about 0.063 g kg^−1^ per cropping cycle.^64^ Such small concentrations would likely not be detectable in incubation experiments where infrared gas analyzers are used to determine the evolved CO_2_.^7^ However, the fact that even a 10 g kg^−1^ concentration did not cause ecotoxicity or DNA damage further supports the hypothesis of the safety of soil‐biodegradable mulch films for mesofauna.

The literature on the effects of *H. illucens* frass on earthworms is notably scarce, and to the best of our knowledge, no studies have specifically addressed this topic. The present study revealed that the concentrations of micronutrients and the chemical composition of the tested frass do not determine earthworms' genotoxicity on coelomocytes cells. However, the phytotoxic effect of frass, especially at high concentrations,^65^ has already been observed, so future studies will be necessary to clarify recommended doses, also considering the diets administered to the insects.

The non‐genotoxic behavior of *H. illucens* frass could be explained by the fact that this organic amendment provides some nutrients, such as carbon and nitrogen, in percentages that are most likely not toxic to *E. fetida*. In fact, it is known that organic matter is vital for the nutrition of earthworms, determining their distribution and abundance.^66^ Furthermore, correct application of nitrogen in the growth substrate is also essential for the wellbeing of earthworms, even if excessive applications could compromise the suitability of these organisms.^67^

### Effects of mulch films, frass and earthworms on soil microbiome

Overall, the treatments involving *H. illucens* frass significantly influenced soil microbial communities, supporting the hypothesis regarding its role in nutrient cycling and soil health. The selective enrichment of bacterial families like Bacillaceae and fungal families like Lichtheimiaceae suggest that the addition of frass promotes microbes with specific ecological functions. Bacillaceae are well known for their role in organic matter decomposition and nitrogen cycling whereas Lichtheimiaceae are associated with lignocellulose breakdown. These findings are consistent with the results of the studies of other studies that demonstrated that insect frass can enhance the activity of fast‐growing decomposer microorganisms.^68^ The presence of insect frass in the soil resulted in an active shaping of the soil microbiome. Moreover, frass contains significant levels of bioavailable nitrogen, phosphorus, and other essential nutrients, as well as chitin, which can act as a stimulant for microbial development. The presence of chitin in frass has been shown to enhance the selection of certain bacterial taxa, such as *Bacillus*, which are capable of breaking down complex organic materials.^69^ This is particularly evident in treatments like soil + EWR + FRS and soil + EWR + SBF + FRS, where Bacillaceae and Actinobacteria were enriched.

The sustained microbial activity, evident from the *β* diversity patterns, suggests that frass creates a nutrient‐dense microenvironment that supports microbial specialization. The results provide evidence supporting this hypothesis, as the enrichment of taxa such as *Streptomyces* and *Thermomyces* in treatments combining frass with SBF indicates an increased microbial potential to degrade complex organic polymers. These microbial taxa are well known for their enzymatic capabilities in polysaccharide metabolism and have been reported previously as active mulch film degraders,^4^, ^70^, ^71^ suggesting that frass can create a favorable environment for the microbial consortia needed for mulch film degradation. The gradual nutrient release from frass is also likely to sustain microbial activity throughout the degradation process.

The addition of SBF presents a unique dynamic within the soil ecosystem. Starch‐based plastics serve as a carbon source for microbial communities, potentially stimulating the growth of specific taxa capable of metabolizing polysaccharides. Interestingly, the combination of SBF with frass and earthworms led to distinct microbial clustering in *β* diversity analyses. Earthworms, such as *E. fetida*, interact with frass to enhance nutrient turnover and microbial activity, and their activity facilitates the aeration and redistribution of frass within the soil matrix, amplifying its effects on microbial communities. This synergistic interaction is evidenced by the clustering of microbial communities in *β* diversity analyses, as treatments containing both earthworms and frass formed distinct groupings. Another study supported this finding, highlighting that earthworms mediate the short‐term recycling of nutrients from organic inputs, improving the efficiency of soil microbial processes.^22^ This may indicate a synergistic effect where frass provides the initial nutrient boost, and SBF acts as a sustained carbon source, creating multiple influences on microbial dynamics.

Soil‐biodegradable mulch films alone did not lead to such a pronounced microbial enrichment but their combination with frass resulted in the development of taxa like *Thermomyces*, highlighting its role in fostering specialized fungal communities. The slow decomposition of SBF may further stabilize these shifts, fostering a microbial environment conducive to long‐term soil health. This finding aligns with previous studies showing that frass applications maintain elevated microbial activity for several weeks, thereby supporting long‐term soil health.^72^

## Conclusion

This study analyzed the effect of the co‐presence of *H. illucens* frass, soil‐biodegradable mulch films, and earthworms in agricultural soil, examining their effects on film biodegradation, ecotoxicity, and soil‐microbiome.

The results show that the biodegradation percentage of the tested film was 9.5% after 22 days, but in the presence of earthworms it increased significantly to 62%. The same film, in the presence of *H. illucens* frass, achieved a biodegradation rate of 52%, suggesting that both earthworms, through soil perturbation, and insect frass, through alteration of the soil microbiota, accelerated the film's biodegradation rate.

The ecotoxicological assessment conducted in this study revealed that soil‐biodegradable mulch film does not cause mortality or induce genotoxic damage in earthworms. Furthermore, the test frass derived from *H. illucens* proved to be harmless to *E. fetida*. Indeed, it even promoted an increase in earthworm biomass, highlighting its potential as a beneficial soil amendment.

The addition of *H. illucens* frass, *E. fetida*, and SBF to soil systems modified microbial community composition and functionality significantly. The two amendments reduced *α* diversity while enriching taxa involved in decomposition and nutrient cycling, demonstrating their action in shaping soil microbiome, enhancing soil fertility. The complementary roles of frass in providing nutrients and stimulating microbial specialization, along with SBF acting as a carbon source, highlight their potential in accelerating the degradation of soil‐biodegradable mulch films. Future research should focus on optimizing application rates and exploring the long‐term impacts of both frass and soil biodegradable mulch films under diverse environmental conditions.

## CONFLICT OF INTEREST

The authors declare no conflict of interest.

## AUTHOR CONTRIBUTIONS

Matteo Francioni: conceptualization, data curation, formal analysis, investigation, methodology, supervision, visualization, writing – original draft. Enrica Marini: conceptualization, data curation, formal analysis, investigation, methodology, visualization, writing – original draft. Arianna De Bernardi: conceptualization, data curation, formal analysis, investigation, methodology, visualization, writing – original draft. Paride D'Ottavio: resources, writing – review and editing. Alessio Ilari: funding acquisition, project administration, resources, writing – review and editing. Ester Foppa Pedretti: funding acquisition, project administration, resources, writing – review and editing. Daniele Duca: writing – review and editing. Boakye‐Yiadom Kofi Armah: writing – review and editing. Chiara Rivosecchi: writing – review and editing. Marco Appicciutoli: formal analysis, investigation. Gianluca Brunetti: writing – review and editing. Marco Bianchini: resources, writing – review and editing. Luigi Ledda: writing – review and editing. Maria Teresa Tiloca: writing – review and editing. Mario Antonello Deroma: investigation, resources. Francesca Tagliabue: writing – review and editing. Cristiano Casucci: writing – review and editing. Costantino Vischetti: writing – review and editing. Filippo Vaccari: data curation, formal analysis, investigation, methodology, visualization, writing – original draft. Francesca Bandini: writing – review and editing. Edoardo Puglisi: writing – review and editing. Paola Antonia Deligios: writing – review and editing.

## Supporting information


**DATA S1.** Supporting Information.

## Data Availability

The data that support the findings of this study are available from the corresponding author upon reasonable request.
