# Supplementary material for: Co‐presence of black soldier fly frass, soil‐biodegradable mulch films, and earthworms: effects on film biodegradation, ecotoxicity, and microbial community
Source: J Sci Food Agric. 2025 Jul 21;105(14):8094–107. doi: 10.1002/jsfa.70064 (PMC12509052; doi:10.1002/jsfa.70064)
Supplement: Supplementary file 1 — DATA S1. Supporting Information. [file JSFA-105-8094-s001.docx]

**Co-presence of Black Soldier Fly Frass, Soil-Biodegradable Mulch Films, and Earthworms: Effects on Film Biodegradation, Ecotoxicity, and Microbial Community**

Matteo Francioni ^1#^, Enrica Marini ^1#^, Arianna De Bernardi ^1*^, Paride D’Ottavio ^1*^, Alessio Ilari ^1^, Ester Foppa Pedretti ^1^, Daniele Duca^1^, Boakye-Yiadom Kofi Armah^1^, Chiara Rivosecchi ^1, 2^, Marco Appicciutoli ^1^, Gianluca Brunetti ^1^, Marco Bianchini ^1^, Luigi Ledda ^1^, Maria Teresa Tiloca ^3^, Mario Antonello Deroma ^3^, Francesca Tagliabue ^1^, Cristiano Casucci ^1^, Costantino Vischetti ^1^, Filippo Vaccari^4*^; Francesca Bandini ^4^; Edoardo Puglisi ^4^; Paola Antonia Deligios ^1^

^1^ Università Politecnica delle Marche, Dipartimento di Scienze Agrarie, Alimentari ed Ambientali, via Brecce Bianche 10, 60131 Ancona, Italy

^2^ Department of Civil, Constructional and Environmental Engineering, Sapienza University of Rome, via Eudossiana 18, 00184 Rome, Italy

^3^ Department of Agricultural Sciences, University of Sassari, Viale Italia 39, 07100 Sassari, Italy

^4^Dipartimento di Scienze e Tecnologie Alimentari per la Sostenibilità della Filiera Agro-Alimentare, Facoltà di Scienze Agrarie Alimentari ed Ambientali, Università Cattolica del Sacro Cuore, Via Emilia Parmense 84, 29122 Piacenza, Italy

#These two authors contributed equally

*correspondence: [p.dottavio@univpm.it](mailto:p.dottavio@univpm.it); [a.debernardi@pm.univpm.it](mailto:a.debernardi@pm.univpm.it); [filippo.vaccari@unicatt.it](mailto:filippo.vaccari@unicatt.it)


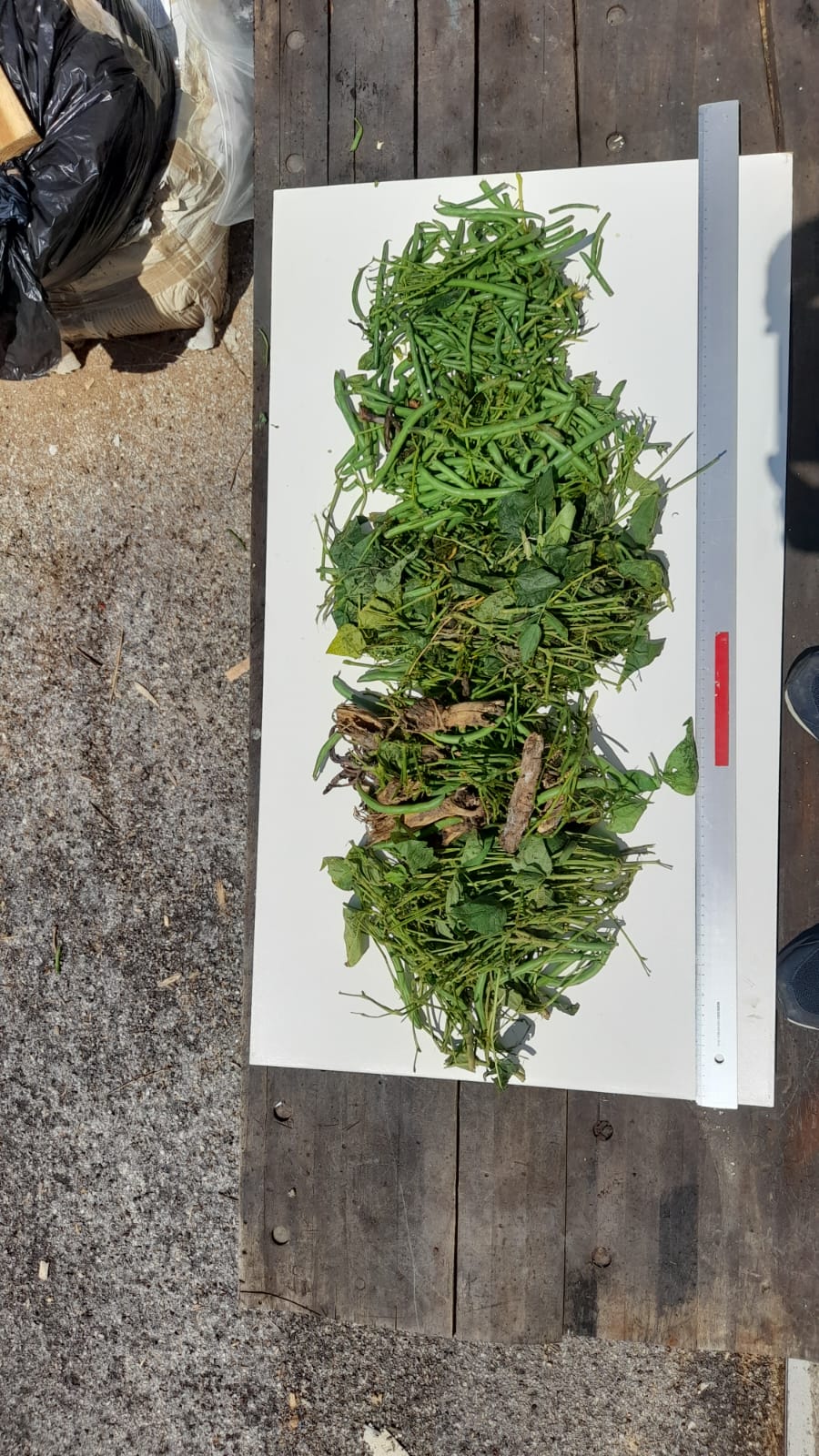


**Figure S1**. Green bean waste from various stages of industrial processing.


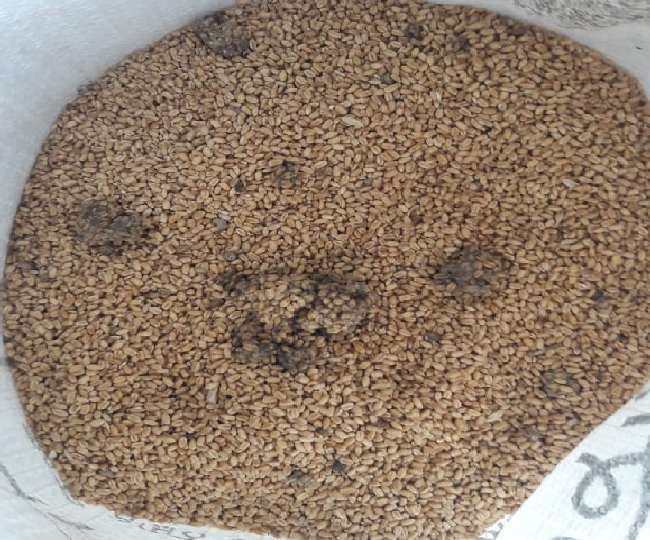


**Figure S2**: Grain storage waste (silo bottom) used for diet formulation.


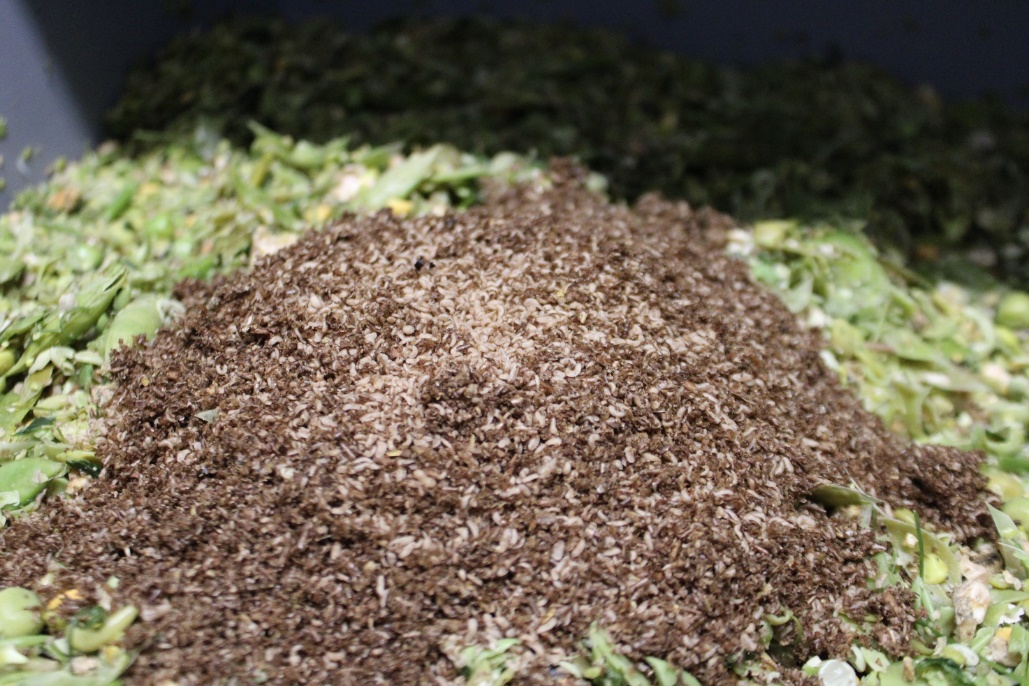


**Figure S3**. Four-six day old larvae fed a diet of green bean and wheat waste.

**DNA amplification and Illumina high-throughput sequencing**

High Througput Sequencing (HTS) of 16S rDNA for bacterial and fungal communities was carried out on soil. The hypervariable V3–V4 region of the bacterial 16S rRNA gene was amplified by PCR to analyze bacterial diversity. The universal primers 343f (5′-TACGGRAGGCAGCAG-3′) and 802r (5′-TACNVGGGTWTCTAATCC-3′) were used. The reaction mix comprised 12.5 μL of Phusion Flash High-Fidelity Master Mix (Thermo Fisher Scientific, Inc., Waltham, MA, USA), 1.25 μL each of forward and reverse primers (10 μM), 1 ng of DNA template, and nuclease-free water to a final volume of 25 μL. The thermal cycling conditions were as follows: initial denaturation at 95 °C for 5 min; 20 cycles of denaturation at 95 °C for 30 s, annealing at 50 °C for 30 s, extension at 72 °C for 30 s; and a final extension at 72 °C for 10 min.

The fungal communities were analyzed using the universal primers ITS-1 (5′-TCCGTAGGTGAACCTGCGG-3′) and ITS-2 (5′-GCTGCGTTCTTCATCGATGC-3′). The PCR reactions required 12.5 µL of Phusion Flash High-Fidelity Master Mix (Thermo Fisher Scientific, Inc., Waltham, MA, USA), 1.25 µL of each primer (10 µM), 2 µL of DNA and nuclease-free water to reach the final volume of 25 µL. The thermocycler was set as follows: an initial hold at 94 °C for 4 min, followed by 28 cycles of 94 °C for 30 s, annealing at 56 °C for 30 s, an extension at 72 °C for 1 min, and a final extension at 72 °C for 7 min. To sequence multiple samples in a single run a two-step PCR was performed, in which first step products were used as a template for the second PCR step, as previously detailed (Berry et al., 2011). Forward primers of the second PCR were indexed with a 9 nucleic acid-base extension at their 5′ as described by (Fontana et al., 2016), and (Vasileiadis et al., 2013). Thermal profiles remained unchanged compared to the first step while the number of cycles for the second amplifications were: 10 cycles for bacterial V3-V4, 10 cycles for archaeal V3-V4.

Products from the second amplification step were multiplexed into a single pool using an equivalent molecular weight of 60 ng per sample. The pool was purified using the solid-phase reversible immobilization (SPRI) method with the Agencourt AMPure XP kit (REF A63880, Beckman Coulter, Milano, Italy). Sequencing was performed by Novogene. (Cambridge, UK) using the TruSeq DNA sample preparation kit (REF 15026486, Illumina Inc., San Diego, CA, USA) for amplicon library preparation. A Novoseq Illumina instrument (Illumina Inc., San Diego, CA, USA) was used to obtain 250 bp paired-end reads.

**Bibliography**

Berry, D., Mahfoudh, K.B., Wagner, M., Loy, A., 2011. Barcoded primers used in multiplex amplicon pyrosequencing bias amplification. Appl. Environ. Microbiol. 77, 7846–7849. https://doi.org/10.1128/AEM.05220-11

Fontana, A., Patrone, V., Puglisi, E., Morelli, L., Bassi, D., Garuti, M., Rossi, L., Cappa, F., 2016. Effects of geographic area, feedstock, temperature, and operating time on microbial communities of six full-scale biogas plants. Bioresour. Technol. 218, 980–990. https://doi.org/10.1016/j.biortech.2016.07.058

Vasileiadis, S., Puglisi, E., Arena, M., Cappa, F., van Veen, J.A., Cocconcelli, P.S., Trevisan, M., 2013. Soil microbial diversity patterns of a lowland spring environment. FEMS Microbiol. Ecol. 86, 172–184. https://doi.org/10.1111/1574-6941.12150

**Table S1** Thermal cycling conditions, primer concentrations and volumes

| **Target** | **Initial denaturation** | **Denaturation** | **Annealing** | **Extension** | **Final Extension** |
| --- | --- | --- | --- | --- | --- |
| V3-V4 | 95° (5 m) | 95° (30 s) | 50° (30 s) | 72° (30s) | 94° (10 m) |
| ITS1-ITS2 | 94° (30 s) | 94° (30 s) | 56° (30 s) | 94° (1 m) | 94° (7 m) |
|  |  | Cycling steps | | |  |

| **Table S2**. Two-way repeated measures ANOVA for CO_2_ emissions. | | | | | |
| --- | --- | --- | --- | --- | --- |
| Source | SS | DF | MS | F | P |
| Tests of within-subjects effects |  |  |  |  |  |
| Time | 0.335 | 6 | 5.59E-02 | 411 | < 0.001 |
| Time × Treatment | 0.246 | 48 | 5.12E-03 | 38 | < 0.001 |
| Error (Time) | 0.015 | 108 | 1.36E-04 |  |  |
| Tests of between-subjects Effects | |  |  |  |  |
| Intercept | 2.385 | 1 | 2.385 | 16569 | < 0.001 |
| Treatment | 0.981 | 8 | 0.123 | 852 | < 0.001 |
| Error (Treatment) | 0.003 | 18 | 1.44E-04 |  |  |

**
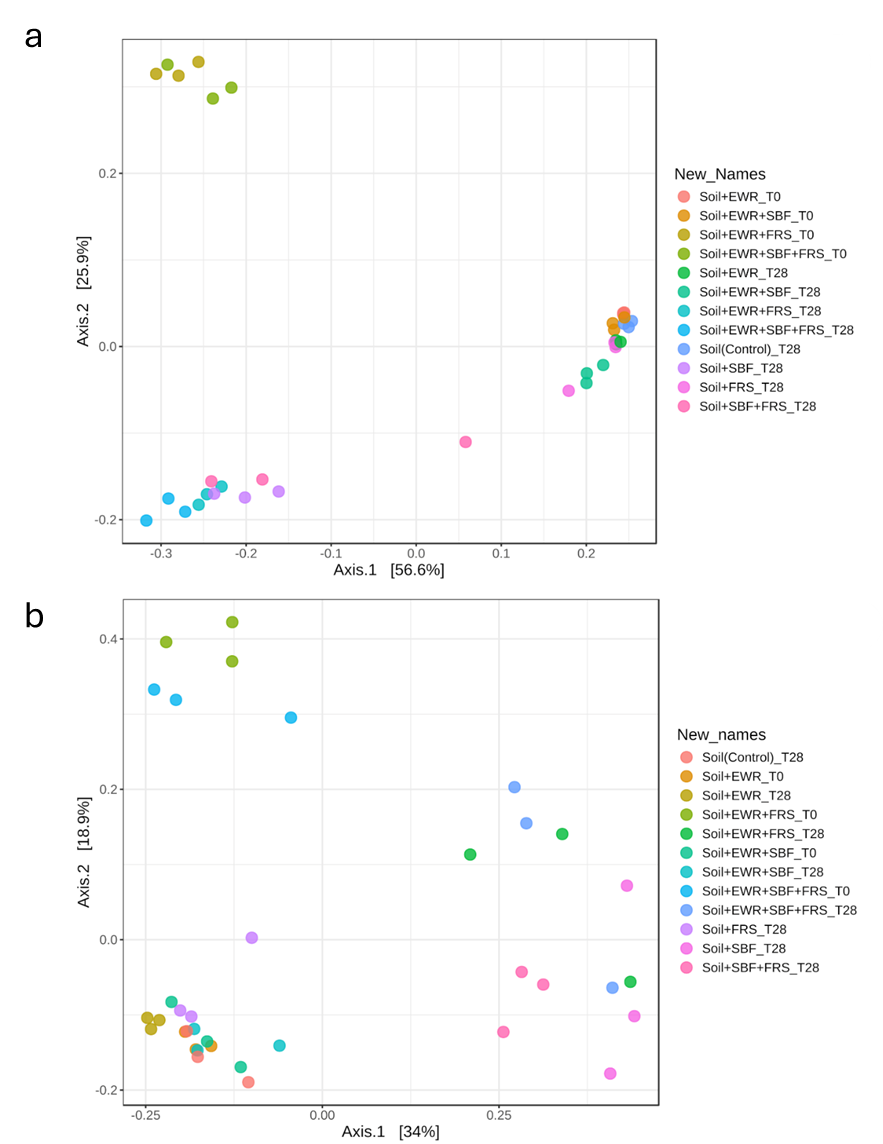
**

**Figure S4**. β-diversity analysis based on two principal coordinates from PCoA of Bray–Curtis distances at the feature level, grouped by treatment. Statistical significance was assessed using PERMANOVA with pairwise comparisons. Panels: (a) bacteria; (b) fungi.


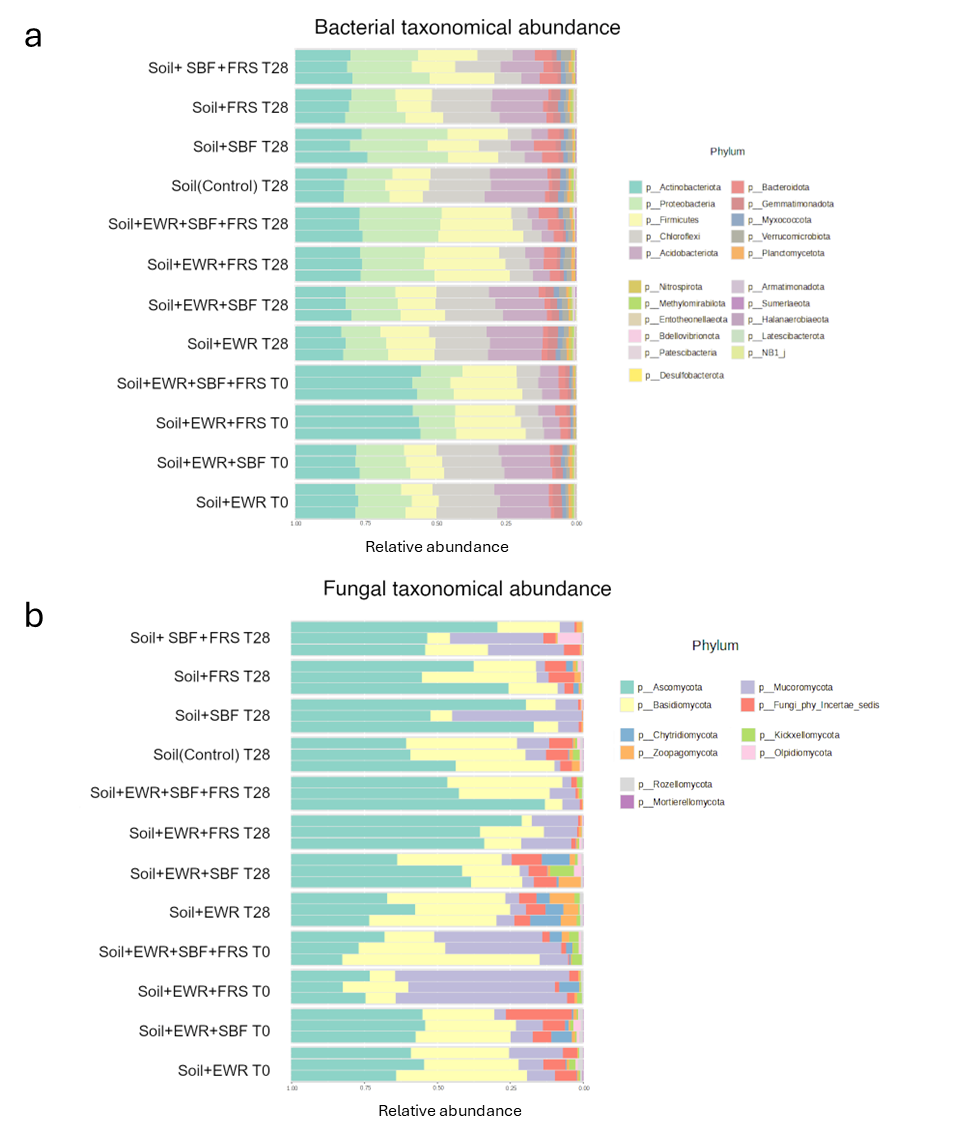


**Figure S5**. Results of amplicon sequencing analysis. Taxa bar plot displaying the relative abundance of microbial phyla for (a) bacteria and (b) fungi across all treatments at two time points: T0 and T28. Treatment names are labelled to the left the bar plots, while the microbial families are listed along the right of the graph.
